# Supplementary material for: Epigenetic control of chromosome-associated lncRNA genes essential for replication and stability
Source: Nat Commun. 2022 Oct 22;13:6301. doi: 10.1038/s41467-022-34099-7 (PMC9588035; doi:10.1038/s41467-022-34099-7)
Supplement: Supplementary file 1 — Supplementary Information [file 41467_2022_34099_MOESM1_ESM.pdf]

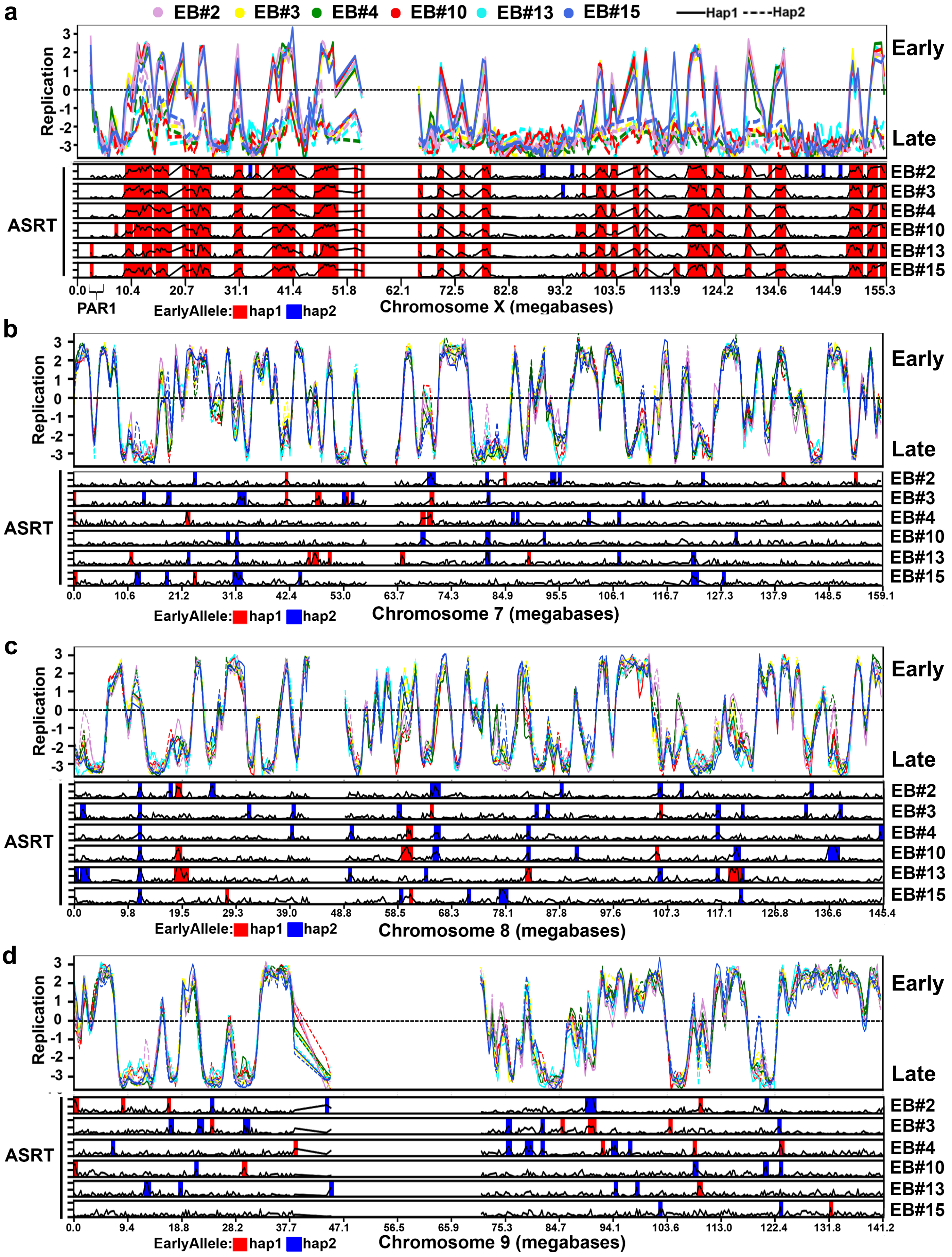

a
EB#2 EB#3 EB#4 EB#10 EB#13 EB#15
Hap1 Hap2
Replication
Early
Late
ASRT
EB#2
EB#3
EB#4
EB#10
EB#13
EB#15
0.0 10.4 20.7 31.1 41.4 51.8 62.1 72.5 82.8 93.2 103.5 113.9 124.2 134.6 144.9 155.3
PAR1
EarlyAllele: hap1 hap2
Chromosome X (megabases)
b
Replication
Early
Late
ASRT
EB#2
EB#3
EB#4
EB#10
EB#13
EB#15
0.0 10.6 21.2 31.8 42.4 53.0 63.7 74.3 84.9 95.5 106.1 116.7 127.3 137.9 148.5 159.1
EarlyAllele: hap1 hap2
Chromosome 7 (megabases)
c
Replication
Early
Late
ASRT
EB#2
EB#3
EB#4
EB#10
EB#13
EB#15
0.0 9.8 19.5 29.3 39.0 48.8 58.5 68.3 78.1 87.8 97.6 107.3 117.1 126.8 136.6 145.4
EarlyAllele: hap1 hap2
Chromosome 8 (megabases)
d
Replication
Early
Late
ASRT
EB#2
EB#3
EB#4
EB#10
EB#13
EB#15
0.0 9.4 18.8 28.2 37.7 47.1 56.5 65.9 75.3 84.7 94.1 103.6 113.0 122.4 131.8 141.2
EarlyAllele: hap1 hap2
Chromosome 9 (megabases)

Figure S1. Asynchronous Replication Timing on human chromosomes. Chromosome Early/Late RT profiles from the 6 EB3_2 clones highlighting regions with SD >1 from the asynchronous replication timing (ASRT) analysis on individual clones. Each clone was color coded as shown, with haplotype 1 shown as a solid line, and haplotype 2 shown as a dotted line for each clone. The left axis shows the RT profiles, with positive numbers representing early replication and negative numbers representing late replication. Early replicating loci with SD >1 are highlighted in red for haplotype 1 and in blue for haplotype 2. The position on each chromosome is shown in magabases. Chromosome profiles are shown for the X chromosome (a), chromosome 7 (b), chromosome 8 (c) and chromosome 9 (d).

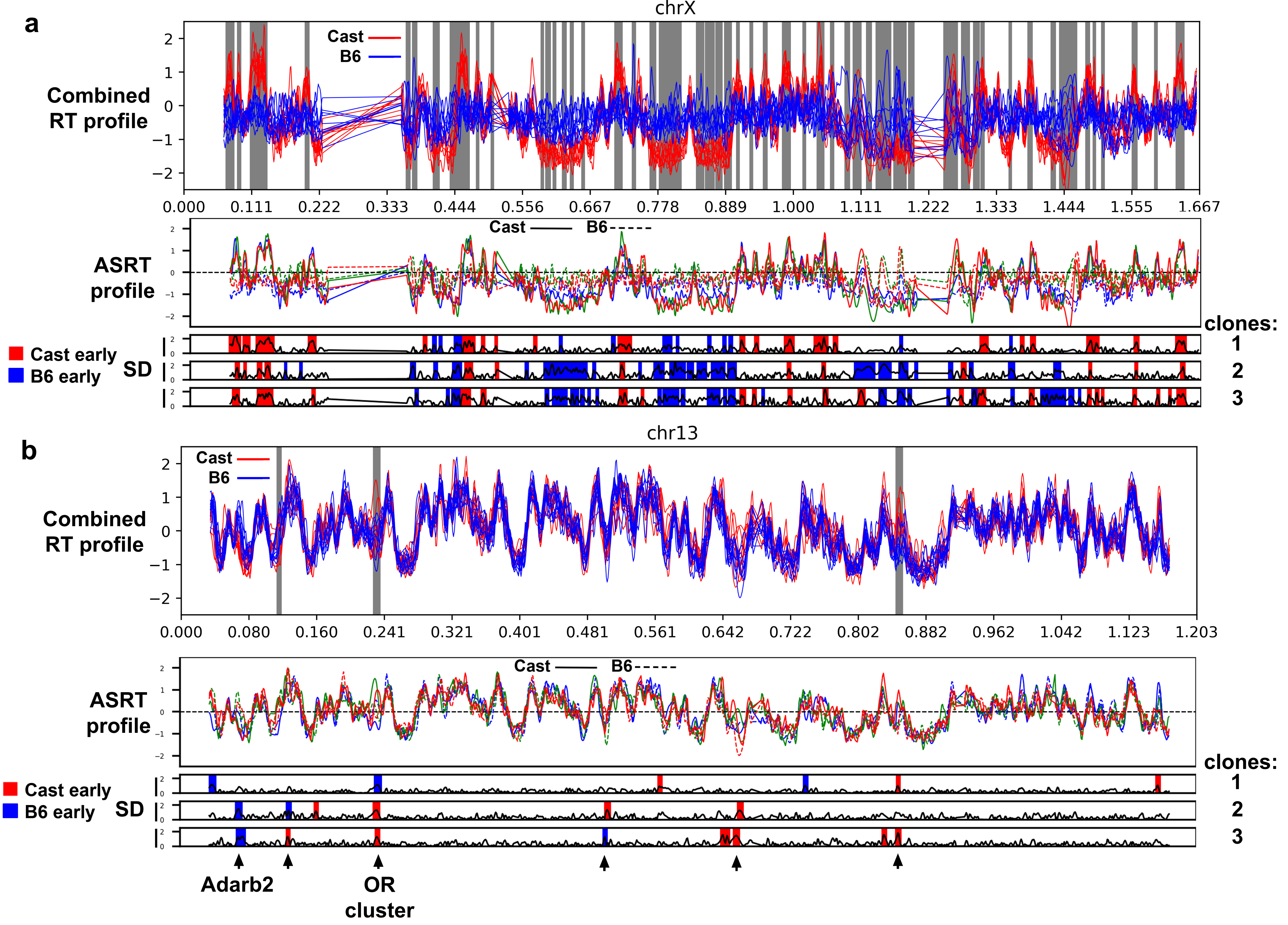
a
chrX
Cast
B6
Combined RT profile
ASRT profile
Cast B6
Cast early
B6 early
SD
clones:
1
2
3
b
chr13
Adarb2
OR cluster

Figure S2. Asynchronous Replication Timing on mouse chromosomes. Chromosome Early/Late RT profiles from 3 pre-B cell clones [from [53]] showing the "combined RT profile" analysis (Top panels) and the asynchronous replication timing profile (ASRT) analysis on individual clones (Middle panels), regions with SD >1 from the ASRT analysis is shown below each panel. For the "combined RT profile" analysis the C57BL6 (B6) allele is blue and the Castaneous (Cast) allele is red, and the regions with SD >1 are highlighted in gray. For the ASRT analysis, each clone was color coded, with the C57BL6 allele as a solid line and the Castaneous allele as a dotted line. The left axis shows the RT profiles, with positive numbers representing early replication and negative numbers representing late replication. From the ASRT analysis early replicating loci with SD >1 are highlighted in red for the Castaneous allele and in blue for C57BL6 allele. The position on each chromosome is shown in magabases. Chromosome profiles are shown for the X chromosome (a) and chromosome 13 (b). The arrows mark 6 loci on chromosome 13 that show ASRT in multiple clones. We detected ASRT at loci that are known to be subject to random monoallelic expression, e. g. an olfactory receptor cluster (OR cluster) and Adarb2 (see Fig. 5a).
