# Supplementary material for: Epigenetic control of chromosome-associated lncRNA genes essential for replication and stability
Source: Nat Commun. 2022 Oct 22;13:6301. doi: 10.1038/s41467-022-34099-7 (PMC9588035; doi:10.1038/s41467-022-34099-7)
Supplement: Supplementary file 2 — Description of Additional Supplementary Files [file 41467_2022_34099_MOESM2_ESM.pdf]

Description of Additional Supplementary Files

File Name: Supplementary Data 1
Description: Strand-specific and allele-specific expression measurements for all TLs and coding genes described within the study in the GM12878 and EB3_2 cell lines. Two-sided binomial test p-values and FDR-BH q-values are included. TLs include the fraction of sequence that is derived from LINE1s elements.

File Name: Supplementary Data 2
Description: Fosmid and BAC clone used in RNA and DNA FISH assays. The Fosmid and BAC clone names (in separate tabs) with the chromosome number, chromosome position (start and stop, in base pairs), locus/gene name, and cell types used are indicated.

File Name: Supplementary Data 3
Description: List of regions with variable epigenetic replication timing between subclones within GM12878 and within EB3_2 cell lines.

File Name: Supplementary Data 4
Description: PCR primers and sgRNA sequences. The names and sequences for each PCR primer and sgRNAs are indicated. Heterozygous SNPs with chromosome number and chromosome position (base pairs) are indicated.

File Name: Supplementary Data 5
Description: List of rtQTL early and late alleles from Koren et al 2014. List of the alleles of EB3_2 and GM12878 and the status of replication timing variability between isogenic subclones (VERT).
