# Supplementary material for: Epigenetic control of chromosome-associated lncRNA genes essential for replication and stability
Source: Nat Commun. 2022 Oct 22;13:6301. doi: 10.1038/s41467-022-34099-7 (PMC9588035; doi:10.1038/s41467-022-34099-7)
Supplement: Supplementary file 8 — Reporting Summary [file 41467_2022_34099_MOESM8_ESM.pdf]

nature portfolio

Corresponding author(s): Mathew James Thayer

Last updated by author(s): 8-24-22

Reporting Summary

Nature Portfolio wishes to improve the reproducibility of the work that we publish. This form provides structure for consistency and transparency in reporting. For further information on Nature Portfolio policies, see our Editorial Policies and the Editorial Policy Checklist.

Statistics

For all statistical analyses, confirm that the following items are present in the figure legend, table legend, main text, or Methods section.

n/a	Confirmed	
☐	☒	The exact sample size (*n*) for each experimental group/condition, given as a discrete number and unit of measurement
☒	☐	A statement on whether measurements were taken from distinct samples or whether the same sample was measured repeatedly
☐	☒	The statistical test(s) used AND whether they are one- or two-sided<br>*Only common tests should be described solely by name; describe more complex techniques in the Methods section.*
☒	☐	A description of all covariates tested
☐	☒	A description of any assumptions or corrections, such as tests of normality and adjustment for multiple comparisons
☐	☒	A full description of the statistical parameters including central tendency (e.g. means) or other basic estimates (e.g. regression coefficient) AND variation (e.g. standard deviation) or associated estimates of uncertainty (e.g. confidence intervals)
☐	☒	For null hypothesis testing, the test statistic (e.g. *F*, *t*, *r*) with confidence intervals, effect sizes, degrees of freedom and *P* value noted<br>*Give P values as exact values whenever suitable.*
☒	☐	For Bayesian analysis, information on the choice of priors and Markov chain Monte Carlo settings
☒	☐	For hierarchical and complex designs, identification of the appropriate level for tests and full reporting of outcomes
☒	☐	Estimates of effect sizes (e.g. Cohen's *d*, Pearson's *r*), indicating how they were calculated

Our web collection on statistics for biologists contains articles on many of the points above.

Software and code

Policy information about availability of computer code

Data collection	Sequencing data collection was performed by firmware on Illumina Sequencing machines (no version available).
Data analysis	All software used for data analysis was based on previously published tools and analyses. Code is available on Github: https://github.com/mheskett/asar_allele_specific<br><br>Software programs and versions:<br>## Alignment: BWA 0.7.17-r1188, STAR 020201<br>## Processing alignments: SAMTools 1.9, BCFtools 1.9, BEDtools v2.27.1<br>## Statistics and plotting: Python 3.6.7, Pandas 0.24.2, NumPy 1.16.2, MatPlotLib 3.1.0

For manuscripts utilizing custom algorithms or software that are central to the research but not yet described in published literature, software must be made available to editors and reviewers. We strongly encourage code deposition in a community repository (e.g. GitHub). See the Nature Portfolio guidelines for submitting code & software for further information.

Data

Policy information about availability of data

All manuscripts must include a data availability statement. This statement should provide the following information, where applicable:

- Accession codes, unique identifiers, or web links for publicly available datasets
- A description of any restrictions on data availability
- For clinical datasets or third party data, please ensure that the statement adheres to our policy

Raw image data are available upon request from the corresponding author MT. The Repliseq and RNAseq sequencing data generated in this study have been deposited in the European Nucleotide Archive database under accession code PRJEB52794 [https://www.ebi.ac.uk/ena/browser/view/PRJEB52794], and are available without restriction.

Human research participants

Policy information about studies involving human research participants and Sex and Gender in Research.

Reporting on sex and gender	N/A
Population characteristics	N/A
Recruitment	N/A
Ethics oversight	N/A

Note that full information on the approval of the study protocol must also be provided in the manuscript.

Field-specific reporting

Please select the one below that is the best fit for your research. If you are not sure, read the appropriate sections before making your selection.

☒ Life sciences ☐ Behavioural & social sciences ☐ Ecological, evolutionary & environmental sciences

For a reference copy of the document with all sections, see nature.com/documents/nr-reporting-summary-flat.pdf

Life sciences study design

All studies must disclose on these points even when the disclosure is negative.

Sample size	For Repli-Seq and RNA-seq analysis sample size was chosen as the maximum number of cell lines available with haplotype resolved genomes and the ability to generate single-cell derived subclones. The number of subclone samples studied in GM12878 and EB3_2 cell lines was chosen as the maximum number of subclones that could successfully be created. The sample sizes are sufficient because each sample was considered independently, and no groupwise or cross sample statistics were needed to test the hypotheses of this study.
Data exclusions	No data exclusions were made.
Replication	All attempts at replication were successful. The general measures taken to verify reproducibility included using replicate slides for each cell type for FISH experiments to validate concordant expression between replicates, and verification that all raw sequence data libraries generated pass quality control metrics.
Randomization	No assignment of patients or samples to groups was used in the research therefore randomization is not applicable.
Blinding	No allocation of patients or samples into groups was used in the research therefore blinding is not applicable.

Reporting for specific materials, systems and methods

We require information from authors about some types of materials, experimental systems and methods used in many studies. Here, indicate whether each material, system or method listed is relevant to your study. If you are not sure if a list item applies to your research, read the appropriate section before selecting a response.

Materials & experimental systems

n/a	Involved in the study
☐	☒ Antibodies
☐	☒ Eukaryotic cell lines
☒	☐ Palaeontology and archaeology
☒	☐ Animals and other organisms
☒	☐ Clinical data
☒	☐ Dual use research of concern

Methods

n/a	Involved in the study
☒	☐ ChIP-seq
☒	☐ Flow cytometry
☒	☐ MRI-based neuroimaging

Antibodies

Antibodies used

Anti-Bromodeoxyuridine-Fluorescein Mouse IgG Clone BMC9318: (Sigma Aldrich: Catalog #11202693001)

Validation

From the Manufacturers website (https://www.sigmaaldrich.com/US/en/product/roche/11202693001): The antibody specifically binds to bromodeoxyuridine and crossreacts with iodouridine (10%). Antibromo- deoxyuridine does not crossreact with fluorodeoxy-uridine, nor with any endogenous cellular components such as thymidine or uridine.
Relevant peer reviewed citations recommended by the manufacturer:
1. Michael Meyer et al.
Journal of cellular and molecular medicine, 24(2), 1774-1785 (2019-12-13).
2. Li-Qiang Tian et al.
Molecular medicine reports, 13(5), 4279-4288 (2016-04-02)
3. Paul Hiebert et al.
Developmental cell, 46(2), 145-161 (2018-07-18)

Eukaryotic cell lines

Policy information about cell lines and Sex and Gender in Research

Cell line source(s)

GM12878 (female EBV transformed Lymphocytes), EB3_2 (female EBV transformed Lymphocytes)
HTD114 cells (male fibrosarcoma). GM12878 and HTD114 were obtained from ATCC. EB3_2 was obtained directly from the author of the publication:
Lajugie, J. et al. Complete genome phasing of family quartet by combination of genetic, physical and population-based phasing analysis. PLoS One 8, e64571 (2013).

Authentication

GM12878 and EB3_2 cells were fully haplotype resolved using published whole genome sequencing data.
HTD114 cells have been extensible genotyped using SNP micro arrays, and we validated numerous SNPs using PCR and Sanger sequencing.

Mycoplasma contamination

None of the cells used in this study were tested for mycoplasma

Commonly misidentified lines
(See ICLAC register)

None of the cells are commonly misidentified lines
